# Evaluation of the Efficacy of Low-Dose Botulinum Toxin Injection Into the Masseter Muscle for the Treatment of Nocturnal Bruxism: A Randomized Controlled Clinical Trial

**DOI:** 10.7759/cureus.32180

**Published:** 2022-12-04

**Authors:** Zaed Ghassan Shehri, Issam Alkhouri, Mohammad Y Hajeer, Ibrahim Haddad, Mohamad Husam Abu Hawa

**Affiliations:** 1 Department of Oral and Maxillofacial Surgery, University of Damascus Faculty of Dentistry, Damascus, SYR; 2 Department of Orthodontics, University of Damascus Faculty of Dentistry, Damascus, SYR; 3 Department of Basic Sciences, University of Damascus Faculty of Dentistry, Damascus, SYR

**Keywords:** teeth grinding, electromyography, emg, masseter muscle, botulinum toxin, gnashing of teeth, nocturnal bruxism, sleep bruxism

## Abstract

Introduction

Botulinum toxin (Botox®) is considered an effective treatment for nocturnal bruxism when injected into the masseter muscle. Several studies have used different dosages of Botox for this purpose. The objective was to determine whether 10 MU of botulinum toxin type A (BTXA) injections into the masseter muscle could lessen nocturnal bruxism.

Material and methods

The sample consisted of 22 patients who suffered from pain in the masseter muscle and sensitivity of the teeth as a result of its wear due to nocturnal bruxism. The sample was randomly divided into two groups. The Botox (BO) group included 11 patients injected with 10 MU of BTXA, and the placebo (PL) group included 11 patients who received a sham intervention. Pain perception was assessed on visual analogue scales, whereas muscle activity was recorded by electromyography (EMG) to evaluate the effectiveness of this treatment protocol on nocturnal bruxism.

Results

A total of 20 patients entered data analysis with one dropout from each group. The differences in the perceived pain values between the BO and the PL groups before and after the injection were statistically significant (p<0.05). In the BO group, the changes in the perceived pain values over time were statistically significant (p<0.05). The pain levels significantly decreased at two weeks, one month, and three months following the injection. However, the levels increased again at the fourth- and sixth-month assessment times with statistically significant differences (p>0.05). The differences in the EMG recorded values were statistically significant between the two groups (p<0.05).

Conclusions

Within the current study's limitations, injecting 10 MU of BTXA into the masseter muscle reduced muscular activity in this muscle, resulting in decreased muscle spasms and pain symptoms associated with nocturnal bruxism for about three months before symptoms gradually relapsed.

## Introduction

Sleep bruxism (SB) is a condition of maxillomandibular activity characterized by nonfunctional teeth grinding and clenching while sleeping [1,2]. Bruxism has been linked to anxiety, stress, and depression, as well as personality types, nutritional deficiencies (magnesium, calcium, iodine, and vitamin complexes), poor dental occlusion, insufficient dental manipulation, central nervous system disorders, use of drugs with neurochemical action, deficient oral proprioception, and genetic factors [3].

In addition to neck pains, headaches, periodontal disease, oral or facial pain, and possibly tooth loss, SB can lead to teeth attrition, dental prostheses/implants failing, tooth sensitivity, pain in the teeth, jaw, masticatory muscle, and temporomandibular joint (TMJ), as well as other problems [4,5]. The diagnosis of nocturnal bruxism is based on complaints of tooth grinding or clenching, as well as one or more of the following signs: nonfunctional teeth attrition, sounds indicative of bruxism, and jaw muscle pain [6]. Bruxism can lead to both TMJ problems and tooth deterioration. Delaying treatment can sometimes result in luxation and degenerative arthritis of the TMJ [7].

For the treatment of bruxism, many treatment approaches such as occlusal splints, pharmacologic medications such as psychobehavioral therapy or L-dopa, and psychobehavioral therapy have been examined but are not enough evidence to define a standard of reference approach for SB treatment [1].

The exotoxin known as botulinum toxin (Botox®) is generated by the bacterium *Clostridium botulinum*, and it paralyzes muscles by preventing the flow of acetylcholine from cholinergic nerve terminals into the neuromuscular junction [8]. In the last two decades, several studies have been conducted to investigate the efficacy of botulinum toxin type A (BTXA) in reducing nocturnal bruxism, and the results have been promising [9]. These studies have used different doses of Botox ranging from 20 mouse units (MU) [10,11] and 25 MU [12] to 30 MU [13,14] in the masseter. Most of these studies did not take into account the relationship between the amount of Botox dose and alteration of the masseter muscle's size and the shape of the lower third of the face, where injection of more than 20 MU into the masseter muscle affects its size and is an effective treatment for masseter muscle hypertrophy for at least nine months [15]. To avoid the unwanted side effects of doses greater than 20 MU, the trial aimed to evaluate the effectiveness of injecting 10 MU of Botox into the masseter muscle in reducing the nocturnal bruxism.

## Materials and methods

Study design and settings

A parallel-group randomized controlled trial study was performed at the Department of Oral and Maxillofacial Surgery, University of Damascus Faculty of Dentistry, Syria, between March 2021 and September 2021. The study was approved by the Local Research Ethics Committee of the Faculty of Dentistry, Damascus University (UDDS-722-10072018/SRC2202). The study was funded by the University of Damascus Postgraduate Research Budget (Reference number: 80015489477DEN), and this was registered at Clinical Trials.gov (ID: NCT05620316).

Sample size calculation

The sample size was calculated using G*power 3.1.7 software (Universität Düsseldorf, Düsseldorf, Germany). The smallest clinically significant difference requiring detection between the two groups on a visual analogue scale (VAS) was assumed to be 20 mm. This variable's standard deviation in a prior study was 11.9 mm [14]. Ten patients were needed in each group using a paired t-test with a significance level of 5% and a power of 80%. With a total sample size of 22 patients, one patient was added to each group to compensate for potential dropouts.

Patients recruitment

The Departments of the Faculty of Dentistry at Damascus University referred 80 patients with bruxism symptoms to the Department of Oral and Maxillofacial Surgery. A questionnaire was given to these patients (Appendix 1) based on a previous relevant study [7], and their answers were taken. The principal investigator (Z.S.) reviewed the patients' answers to the questionnaire. A total of 65 patients were found to have active bruxism. Then, they were clinically examined by the principal investigator (Z.S.). The inclusion criteria were met by 32 patients. The inclusion criteria were as follows: (1) pain in the masseter muscles during clinical examination classified moderate to severe, (2) age between 18 and 40 years, (3) tooth-grinding sounds confirmed by family members or caregivers, and (4) attrition in occlusal surface of posterior teeth. Exclusion criteria were as follows: (1) loss of two posterior teeth or more (except for third molars), (2) fixed or movable prosthodontics for more than four dental units, (3) advanced malocclusion (class II occlusion model II - deep bite - open bite), (4) temporomandibular disorders, (5) pain in the orofacial region, (6) insomnia, (7) known Botox allergy, (8) pregnancy, (9) diseases that affect neuromuscular, (10) diseases related to hemorrhage, (11) antibiotic-based treatment, (12) the site of the injection is infected with an infectious lesion, and (13) respiratory ailments that caused nighttime coughing.

**Figure 1:**
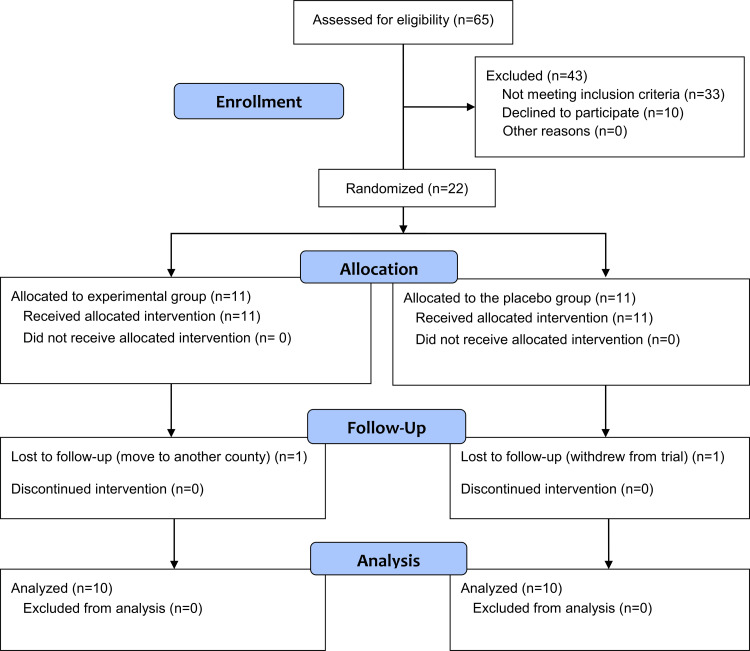
The Consolidated Standards of Reporting Trials (CONSORT) flow diagram of patients' enrollment, allocation, follow-up, and inclusion into data analysis.

All candidate patients were informed about the research project's purpose and given information sheets. Informed consent was obtained from those who agreed to participate. A total of 28 people consented to participate in the current study. The a priori sample size calculation revealed the need for 22 patients for this trial. A simple random sampling was employed to select these 22 patients out of the 28 individuals who had been found eligible for this trial and accepted participation (i.e., from the sampling frame). They were then randomly divided into the experimental group (BO; n=11) and the placebo group (PL; n=11) with a one-to-one allocation ratio. The following information was recorded before the intervention: treatment history of nocturnal bruxism and duration of the complaint. The flow diagram for patient recruiting, follow-up, and entry into data analysis according to the Consolidated Standards of Reporting Trials (CONSORT) is given in Figure 1.

Randomization and allocation concealment

With a 1:1 allocation ratio, computer-generated random numbers were used to perform the randomization. The allocation sequence was hidden with numbered, opaque, and closed envelopes that were opened before treatment began. The patients were randomly allocated into two groups. In the BO group, the patients received BTXA, whereas in the PL group, the patients received a placebo. An academic staff member not involved in the study project was asked to perform the random allocation sequence and participants' enrollment. The opaque sealed envelopes bearing the patient's name's initials were used to conceal the allocation sequence. The envelope was opened just before the injection for each patient. The first author (Z.S.) has treated patients and was aware of which group each patient belonged to. Blinding of the principal researcher was not applicable. Therefore, blinding was applied only to the patients and during data acquisition and analysis.

The experimental group: the Botox group

**Figure 2:**
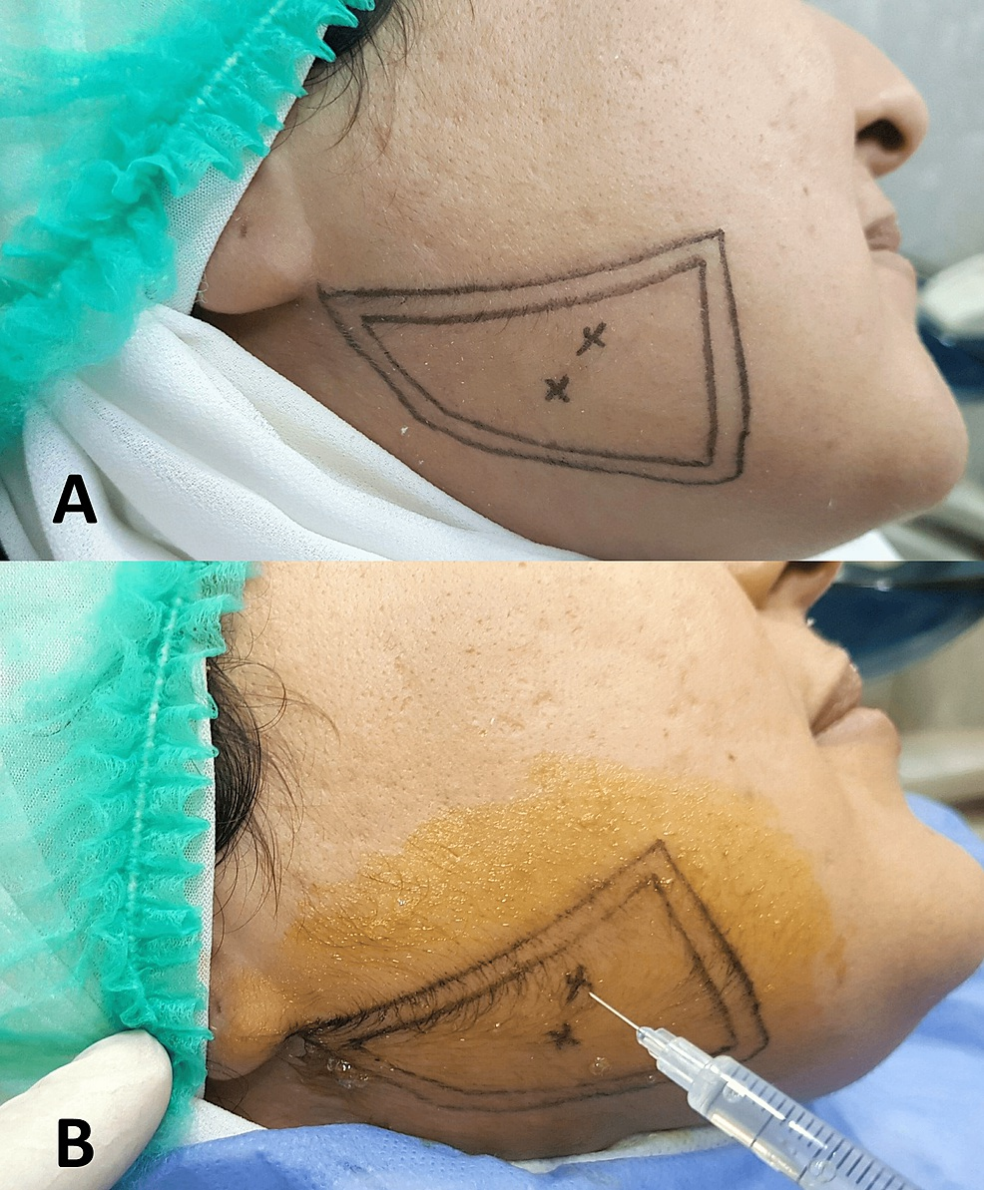
(A) Determination of the area of injection. (B) Two points were used for injecting 10 MU of botulinum toxin type A.

In this group, 100 MU of Botox type A (BOTOX®, Allergan Inc., Dublin, Ireland) were diluted in 2 mL of saline. Patients were injected with 10 MU of BTXA per side at two sites into masseter muscle bilaterally. The first site was the inferior prominent part of the masseter muscle observed when the subject was asked to clench, and the other site was 5 mm below the first point (Figure 2).

The placebo group

In this group, patients were pricked twice at the inferior prominent part of the masseter muscle observed using the stinger pen (SOFTCLIX II, Accu-Chek, Basel, Switzerland) used in the blood glucose meter. It is less painful and provides psychological benefits, instead of injecting the physiological saline into the muscle to avoid the severe pain without a benefit to the patient, which would not conform to the ethical standards.

Outcome measures

Overall Assessment

The following information was recorded two weeks after the injection: (1) when the effects were first seen by patients and (2) the side effects of Botox injections. In addition, the time when the patients felt that the effectiveness of the injection started to be lost was recorded at two assessment times: three and four months following the intervention.

Pain Assessment

The pain was measured using a VAS. The patient was asked to mark on a line that measured 100 mm in length where she/he reflected her/his perceived pain. The scores of the scale were determined by measuring the distance in mm from the beginning to the point indicated by the patient (point (0): no pain and point (100): the highest levels of pain). This assessment was made before the injection (T0) and then at two weeks (T1), one month (T2), three months (T3), four months (T4), and six months (T5) after the injection.

Electromyography (EMG) Analysis

Electromyography (EMG) signals were recorded with Matrix EP Light EMG (Micromed, Via Giotto, Mogliano Veneto, Italy) with two channels. The electrodes were placed on the belly of the right and left masseter muscles, with a larger extension of bars perpendicular to the direction of muscle fibers [16]. A circular electrode was placed on the forehead as a reference electrode (ground electrode). Before placing the electrodes, the skin region was cleaned with alcohol 70% and shaved to reduce the impedance.

The recorded signals were amplified and sampled at 1024 Hz, and the acquired data were analyzed with System Plus Evaluation software (Micromed). The acquisitions were performed twice with the rest position of the mandible (RPM) for 10 seconds, in maximal intercuspal position (MIP) for 5 seconds, and maximal teeth clenching (MTC) with 10-mm thick cotton rolls between the posterior teeth for 5 seconds, bilaterally, and the values obtained were averaged [17]. The unit of measurement used in the EMG records was microvolts (µV). This assessment was made before the injection (T0) and then at two weeks (T1), three months (T2), and six months (T3) after the injection.

Statistical analysis

SPSS software (version 24, SPSS Inc., Armonk, NY, USA) was used for the statistical analysis. The Kolmogorov-Smirnov test was used to check the normality of the distributions. Independent t-tests were used to detect significant differences between the two groups. A p-value of <0.05 was considered statistically significant.

## Results

Basic sample characteristics

The sample consisted of 20 patients (13 females and seven males) aged 18 to 40 years. The average age of the recruited sample was 29.81±7.12 years (30.23±7.65 years for males and 29.57±6.83 for females). The mean duration of the complaint before injection was 7.33±4.85 years (Table 1). The mean time that the effects were first seen was 10.82±1.93 days.

**Table 1:** Basic sample characteristics (age, sex, and duration of complaint) N, number of patients; SD, standard deviation

Variable	Botulinum toxin (N=10)	Placebo (N=10)	Both groups (N=20)
Age: mean ± SD (years)	29.64±6.75	30.21±7.3	29.81±7.12
Duration of complaint: mean + SD (years)	6.73± 6.1	8.27±5.38	7.33±4.85
Sex, N (%)			
Male	3 (30%)	4 (40%)	7 (35%)
Female	7 (70%)	6 (60%)	13 (65%)

Pain scores

The BO group's mean VAS score was 8.62 mm (±1.35) at T0 before injection. After two weeks (T1) and one month (T2) of the injection, it decreased to 2.83 mm (±1.47) and 1.32 mm (±1.33), respectively (Table 2), and the differences were statistically significant (p<0.001, at T1 and T2; Table 3). After three (T3), four (T4), and six months (T5), it increased to 2.41 mm (±1.97), 4.73 mm (±1.05), and 6.07 (±1.05), respectively, and the differences were statistically significant (P=0.037, p<0.001, and p=0.022, at T3, T2, and T5, respectively).

**Table 2:** Descriptive statistics of the visual analogue scale scores for the two groups at the six assessment times and the p-values of significance testing. *An independent-samples t-test was used to detect significant differences in the visual analogue scale scores between the two groups. BO, Botox group; PL, placebo; SD, standard deviation; Min, minimum value; Max, maximum value

Assessment time	Group	Mean	SD	Min.	Max.	P-value*
Before injection	BO	8.62	1.35	7.53	9.82	0.857
	PL	8.51	1.08	7.24	9.53	
2nd Week	BO	2.83	1.47	1.65	5.06	<0.001
	PL	8.04	1.05	6.73	9.24	
1st Month	BO	1.32	1.33	0.76	4.65	<0.001
	PL	8.07	1.15	6.58	9.86	
3rd Month	BO	2.41	1.97	0.96	4.92	<0.001
	PL	8.42	0.99	6.23	9.57	
4th Month	BO	4.73	1.05	3.65	6.38	<0.001
	PL	8.34	0.67	7.16	9.66	
6th Month	BO	6.07	1.05	4.97	7.53	<0.001
	PL	8.62	0.51	6.98	9.84	

**Table 3:** Comparisons of the mean values of pain perception in the Botox group between different assessment times with p-values of significance testing. *A paired-samples t-test was used to detect significant differences in the visual analog scale scores between the assessment times in the Botox group. SD, standard deviation

Comparison between assessment times	Mean	SD	P-value*
Before injections vs. 2nd week			
Before injection	8.62	1.35	<0.001
2nd Week	2.83	1.47	
2nd Week vs. 1st month			
2nd Week	2.83	1.47	<0.001
1st Month	1.32	1.33	
1st Month vs. 3rd month			
1st Month	1.32	1.33	0.037
3rd Month	2.41	1.97	
3rd Month vs. 4th month			
3rd Month	2.41	1.97	<0.001
4th Month	4.73	1.05	
4th Month vs. 6th month			
4th Month	4.73	1.05	0.022
6th Month	6.07	1.05	

The PB group's mean VAS score was 8.51 mm (±1.08) at T0 before injection. After two weeks of the injection, it decreased slightly to 8.04 mm (±1.05). During the follow-up periods, the mean VAS scores were 8.07 mm (±1.15), 8.42 mm (±0.99), 8.34 mm (±0.67), and 8.62 mm (±0.51) at T2, T3, T4, and T5, respectively.

The mean pain scores were similar between the two groups before injection (T0) without statistically significant differences (p=0.8574). The mean pain scores were smaller in the BO group at T1, T2, T3, T4, and T5, and the differences between the two groups were statistically significant (p<0.001).

EMG recording

In the BO group, the mean EMG records of muscular activity before the injection were 596.38 Microvolt (µV) and 694.77 (µV) for the right and left masseter muscles, respectively. After two weeks (T1) of the injection, the mean values decreased to 258.80 µV and 328.84 µV for the right and left masseter muscles, respectively. After three months (T2), the mean values were 257.26 µV and 336.05 µV for the right and left masseter muscles, respectively. After six months (T3), the mean values increased to 539.91 µV and 700.61 µV for the right and left masseter muscles, respectively (Tables 4, 5).

**Table 4:** Comparison of EMG records of muscular activity for the right masseter between the two groups according to the assessment time. *An independent-sample t-test was used to detect significant differences in EMG records of muscular activity for the right masseter (in µV) between the two groups. BO, Botox group; PL, placebo; SD, standard deviation

Assessment time	Group	Mean	SD	P-value*
Before injection	BO	596.38	363.02	0.645
	PL	533.09	226.19	
2nd Week	BO	258.80	160.40	0.013
	PL	515.56	248.13	
3rd Month	BO	257.26	160.83	0.015
	PL	528.66	274.92	
6th Month	BO	539.91	316.18	0.789
	PL	574.61	251.81	

**Table 5:** Comparison of EMG records of muscular activity for the left masseter between the two groups according to the assessment time. *An independent-sample t-test was used to detect significant differences in EMG records of muscular activity for the left masseter (in µV) between the two groups. BO, Botox group; PL, placebo; SD, standard deviation

Assessment time	Group	Mean	SD	P-value*
Before injection	BO	694.77	360.76	0.760
	PL	645.83	193.89	
2nd Week	BO	328.84	181.01	0.003
	PL	620.07	205.86	
3rd Month	BO	336.05	188.57	0.008
	PL	623.69	236.12	
6th Month	BO	700.61	387.83	0.736
	PL	647.76	233.73	

In the PB group, the mean EMG records of muscular activity at T0 were 533.09 µV and 645.83 µV for the right and left masseter muscles, respectively. After two weeks (T1), the mean EMG records were 515.56 µV and 620.07 µV for the right and left masseter muscles, respectively. After three months (T2), the mean EMG records were 528.66 µV and 623.69 µV for the right and left masseter muscles, respectively. After six months (T3), the mean EMG records increased to 574.61 µV and 647.76 µV for the right and left masseter muscles, respectively.

The mean EMG records of muscular activity were statistically smaller in the BO group compared to the PB group at T1 (p=0.013 and p=0.003 for the right and left masseter muscles, respectively) and T2 (p=0.015 and p=0.008 for the right and left masseter muscles, respectively). The mean EMG records of muscular activity became similar between the two groups at T3 (p>0.05).

Loss of effectiveness and side effects

The mean time at which the loss of effectiveness started was 3.51±0.36 months. Regarding complications, four patients had pain in the injection points in the first week of injection, and two patients had discomfort at the injection site. No side effects were reported by the rest of the included patients.

## Discussion

The management of nocturnal bruxism is usually directed at protecting teeth, restorations, and reducing bruxism and pain relief through stress control, occlusal balancing, occlusal splint, and drugs [18]. Botox was first used to manage nocturnal bruxism in a patient with brain injury by Van Zandijcke and Marchau in 1990 [19].

Because the masseters are the main muscles implicated in the grinding actions seen in bruxism [20], they were injected in this study. The remaining masticatory muscles (temporalis, medial and lateral pterygoid, digastric, and geniohyoid) were not treated for chewing and swallowing to take place. This differs from earlier reports of successful bruxism treatment involving injections into the masseter and temporalis muscles [21,22]. Simply injecting masseter muscle has therapeutic advantages, likely due to a decrease in peripheral muscle activity without affecting the central nervous system [11].

In our study, we injected 10 MU of BTXA into the masseter muscle and evaluated the efficacy of this dose in reducing nocturnal bruxism. The first major main finding was that, according to the EMG recording, muscular activity significantly decreased in the masseter muscle in the BO group compared with the PL group. Muscle activity was markedly reduced at two weeks after injection and maintained for 12 weeks, and then muscle activity increased after six months and returned to what it was before the injection.

The second main finding was that, according to the VAS scales, the pain values were significantly decreased in the BO group compared with the PL group. However, in the fourth month, pain scores began to increase with the loss of effectiveness of Botox. Moreover, they remained lower than the pain scores in the PL group. This is due to injecting a lower dose of Botox into the masseter muscle.

An injection of 10 MU of BOTOX into the masseter muscle eliminated active bruxism for three months, after which the symptoms resumed, as demonstrated in our study. However, it has been proposed that BTXA-induced jaw muscle paralysis disrupts the feedback loop from the trigeminal motor nucleus, inhibiting the central bruxism generator. In addition, during mastication, it may deactivate periodontal mechanoreceptors, which have been suggested to facilitate jaw closure of motor neurons [23].

These outcomes are in agreement with a previous study by Guarda-Nardini et al. in which 20 patients were studied to determine the effectiveness of Botox and saline solution in reducing pain associated with myofascial pain caused by bruxism. The pain levels were assessed using a VAS in the range of 0-10 at rest and during mastication, before and after the Botox injection. Ten patients received a saline solution, and the remaining 10 patients were injected with 30 MU of Botox type A (BOTOX) at three places in the masseter muscles and 20 MU at two points in the anterior temporalis muscles. The authors reported a significant decrease in pain scores in the injection group compared to the PB group [13]. Another study by Al-Wayli reported that nocturnal bruxism could be treated with injected 20 MU of BTXA into each masseter muscle, reducing pain scores in the injection group during the follow-up period [11].

A study by Lee et al. evaluated the efficacy of BTXA (Dysport) injections in treating nocturnal bruxism. The sample included 12 patients: in six patients, 80 MU of BTXA (Dysport) was injected into the masseter muscle, and in the other patients, saline solution was injected. The nocturnal electrical activity (EMG) of the masticatory and temporal muscles was recorded in a normal sleep environment before and after the injection for 4, 8, and 12 weeks, and VAS was used to measure pain. The study’s finding revealed a decrease in the effectiveness of the masseter muscle activity during sleep, which reduced the occurrence of bruxism [24]. Another study by Shim et al. 2014 evaluated the efficacy of Botox on jaw movements during sleep in bruxism patients who did not respond to occlusal splints. The sample involved 20 patients, ten patients injected 25 MU of Botox into the masseter muscle, and the other patients received injections in both the masseter and temporalis muscle. Video polysomnography (VPSG) was used to record the rhythmic activity of masticatory muscles (RMMA), oral and facial activity (OFA), and EMG in both muscles before injection and after four weeks. The authors found that Botox injection did not reduce the frequency and number of pulses and duration of (RMMA) attacks in both groups, while it reduced the intensity of muscle contraction injected during sleep [12].

We agreed with the results of these previous studies. Whereas Botox type A blocks acetylcholine release from cholinergic nerve terminals into the neuromuscular junction, reducing muscular activity [25] and temporary paralysis of the masseter muscle, this could be beneficial in reducing nocturnal bruxism [26]. Muscle activity recovers after six months due to the removal of Botox’s action, as the nerve endings re-establish their original synaptic connection and the neuromuscular connection re-establishment [27].

In addition, it was noted that four patients experienced injection site pain for a week following the injection, and two patients had discomfort at the injection site, with no other Botox injection side effects. These outcomes agreed with a previous study by Park et al.2003 on the occurrence of injection site pain and disagreed with it in the presence of chewing difficulties, speech disturbance, and abnormal facial appearance [28]. At the same time, These outcomes disagree with the findings of Shim et al. and Al-Wayli, who found no evidence of Botox injection-related side effects [11,12].

Limitations

The current study had some limitations. First, blinding the researcher was not possible, which could have produced a detection bias. Secondly, there was no attempt to evaluate the outcomes following temporal muscle injection. Additionally, differences between the two genders were not assessed. Patients' psychosocial factors that may have affected the degree of bruxism were not evaluated in the current trial. This can be avoided in future studies by a pre-intervention psychological analysis of all possible contributing factors.

## Conclusions

Within the limitations of the current study, injecting 10 MU of Botox into the masseter muscle reduced muscle activity and pain symptoms associated with nocturnal bruxism. This effect lasted for about three months, and then the symptoms gradually returned.

## Figures and Tables

**Figure 3:**
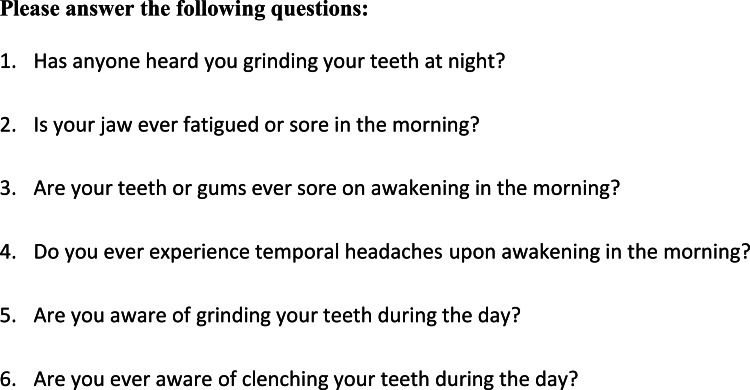
Appendix 1: The questionnaire used in the screening stages of patient recruitment.
